# Focused VHEE (very high energy electron) beams and dose delivery for radiotherapy applications

**DOI:** 10.1038/s41598-021-93276-8

**Published:** 2021-07-07

**Authors:** L. Whitmore, R. I. Mackay, M. van Herk, J. K. Jones, R. M. Jones

**Affiliations:** 1 Department of Physics and Astronomy, University of Manchester, Manchester, UK; 2 The Cockcroft Institute of Science and Technology, Daresbury, Warrington, UK; 3 The Christie NHS Foundation Trust, Manchester Academic Health Science Centre, Manchester, UK; 4 Division of Cancer Sciences, School of Medical Sciences, Faculty of Biology, Medicine and Health, The University of Manchester, Manchester, UK; 5 ASTeC, STFC Daresbury Laboratory, Daresbury, Warrington, UK

**Keywords:** Radiotherapy, Physics, Particle physics

## Abstract

This paper presents the first demonstration of deeply penetrating dose delivery using focused very high energy electron (VHEE) beams using quadrupole magnets in Monte Carlo simulations. We show that the focal point is readily modified by linearly changing the quadrupole magnet strength only. We also present a weighted sum of focused electron beams to form a spread-out electron peak (SOEP) over a target region. This has a significantly reduced entrance dose compared to a proton-based spread-out Bragg peak (SOBP). Very high energy electron (VHEE) beams are an exciting prospect in external beam radiotherapy. VHEEs are less sensitive to inhomogeneities than proton and photon beams, have a deep dose reach and could potentially be used to deliver FLASH radiotherapy. The dose distributions of unfocused VHEE produce high entrance and exit doses compared to other radiotherapy modalities unless focusing is employed, and in this case the entrance dose is considerably improved over existing radiations. We have investigated both symmetric and asymmetric focusing as well as focusing with a range of beam energies.

## Introduction

Radiotherapy is used in the treatment of around half of all cancer cases worldwide^1^, and 27% of cases in the UK^2^. The aim of radiotherapy is to deliver a lethal dose to the tumour whilst sparing healthy tissue as much as possible; since it was first invented the field has advanced greatly and highly conformal photon radiotherapy techniques such as intensity modulated radiotherapy (IMRT) and volumetric modulated arc therapy (VMAT) are now routinely used in cancer treatment^3^. In addition to conventional radiotherapy, FLASH radiotherapy in which ultra-high dose rates are used to deliver radiotherapy in a manner that reduces normal tissue toxicity is currently being investigated^4^ with the first patient treated in Lausanne in 2019^5^. Proton radiotherapy is becoming more widely used due to the highly favourable dose distribution in the form of the Bragg peak, with minimal dose delivered downstream from the target in the beam direction. Proton radiotherapy has the disadvantage of requiring large halls and expensive accelerators and beam transport systems^6^; as such there is current interest in other forms of radiotherapy that have similarly beneficial dose distributions but are less space-intensive.

**Figure 1:**
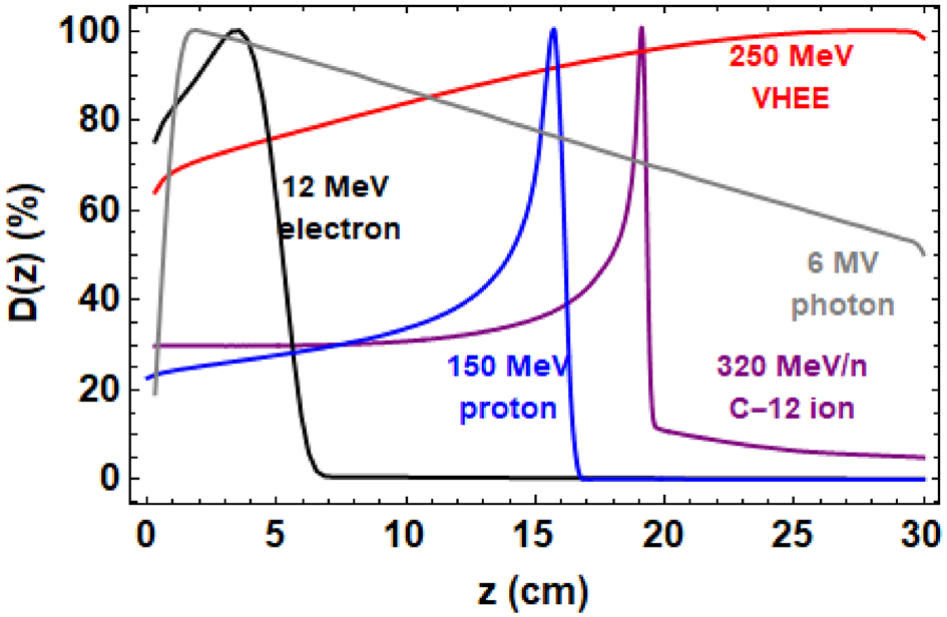
TOPAS-based Monte Carlo simulations of the integrated normalised dose deposited in the plane parallel to the direction of an incident Gaussian beam ($\sigma =4$ mm) for 6 MV photons, 12 MeV electrons, 150 MeV protons, 320 MeV/n carbon-12 ions and a 250 MeV VHEE beam. All beams are in the absence of focusing.

For deep-seated tumours, the radiation must penetrate 15–30 cm into the body. Different types of radiotherapy have different integrated dose distributions, as shown in Fig. 1.

Conventional electron radiotherapy peaks at or before 5 cm into the body before sharply falling off, making it unsuitable for the treatment of deep-seated tumours. Clinical photon beams also sharply peak close to the surface of the patient before exponentially falling off, resulting in a high entrance dose, though this can be decreased by combining multiple beams from different angles. Proton beams can penetrate far into the patient and deposit most of their dose in the Bragg peak before falling off sharply, and have a relatively low entrance dose, although this does increase up to $\ge$40% of the maximum dose for higher energy ($\ge$180 MeV) proton beams. The entire tumour region can be covered using multiple proton beams of different energies weighted by intensity using for example the method shown in the 1984 paper by Bortfeld and Schlegel^7^ in order to produce a spread-out Bragg Peak, although this results in a significantly higher entrance dose (60–80%). Proton beams are highly affected by inhomogeneities^8^, with the result that a small change in composition in the body pre-treatment such as the presence of an air gap can result in the proton Bragg peak being deposited outside of the tumour. Motion due to for example breathing can also cause the Bragg peak to be deposited in the wrong region. Proton radiotherapy therefore requires robustness measures which increase the dose close to the target. Carbon-ion beams have similar dose distributions and characteristics to proton beams, but with reduced penumbral spreading^9^, a fragmentation ’tail’ after the Bragg peak, and also a smaller percentage energy spread^10^ resulting in a sharper Bragg peak. Carbon-ion beams also have a higher relative biological effectiveness (RBE) thereby causing more damage to the tumour, but with the disadvantage of requiring higher energy machines, which are more expensive and require significantly more space than proton radiotherapy machines^11^.

Very high energy electron (VHEE) beams (with energies in the region 50–250 MeV) can penetrate far into the body. The benefit of this is that VHEE beams could be used to treat deep-seated tumours and have been shown to be much less sensitive to inhomogeneities than proton beams^12^. While no suitable machines explicitly developed for VHEE radiotherapy currently exist, advancements in accelerator technology have enabled linear accelerators (linacs) to robustly produce 100 MeV/m gradients^13–15^. This provides some confidence that similar robust structures could be used for medical linacs^16^. This combined with the fact that VHEE beams could be delivered rapidly makes VHEE an attractive candidate for FLASH radiotherapy^16–18^. There are currently plans for two VHEE FLASH radiotherapy treatment machines, the CERN/CHUV collaboration^16^ and PHASER at Stanford University^19^. Studies by Bazalova Carter et al.^20^ have shown that VHEE plans are consistently similar or better at treating certain tumours than the photon VMAT plans. However, VHEE dose distributions have a high entrance and exit dose. In order to circumvent this, the possibility of using quadrupoles to focus the VHEE beams before they enter the phantom has been investigated.

Studies by McAuley et al.^21^ using proton beams and by Kokurewicz et al.^22^ and Lagzda et al.^23^ using VHEE beams have shown experimentally that beam focusing at shallow depths of approximately 5 cm (comparable to normal electron beam radiotherapy) in a water phantom is possible using quadrupole magnets. The aim of this study is to investigate the feasibility of using realistic quadrupole magnets to focus deeply penetrating VHEE beams by performing Monte-Carlo simulations to depths $\ge$15 cm. The resultant dose distributions inside a water phantom will be investigated for 250 MeV symmetrically and asymmetrically focused beams, with the asymmetrically focused beams also being investigated for 100 MeV, 150 MeV and 200 MeV. The effect of changing the final magnet strength on the on-axis dose distributions and beam $\sigma$ will also be investigated, both using Monte Carlo and analytical calculations. The dose distribution results will be used to produce a spread-out electron peak over a target region in the water phantom by summing the dose distributions with weighting factors. This will be compared to a proton spread-out Bragg peak (SOBP) over the same region.

## Results

The Elegant particle physics code^24^ is a rapid, well-tested code used routinely in accelerator physics for machine design of particle orbits in a vacuum. The Elegant code is however unable to predict the effects of scattering in air and water; as such Monte-Carlo simulations in TOPAS^25^ are also required to model focused VHEE beams in a water phantom. The Elegant code was used to rapidly find the initial optimal number, position and strengths of quadrupoles to focus 250 MeV beams symmetrically and asymmetrically in a vacuum. This optimisation required multiple iterations that would take many hours using the TOPAS code, compared to seconds in the Elegant code. Thus the initial simulations in Elegant formed a starting basis in the optimised focusing positions in TOPAS simulations. The method and quadrupole strengths used are described in the “Methods” section.

**Figure 2:**
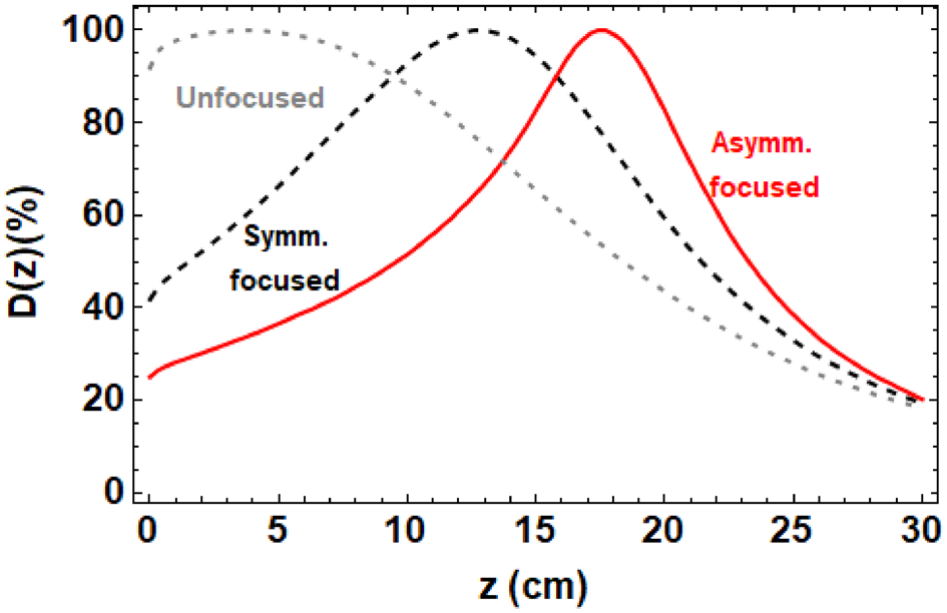
TOPAS-based Monte Carlo simulations of the normalised on-axis dose plots for 250 MeV symmetric and asymmetric focused beams, as well as unfocused 250 MeV VHEE.

The normalised on-axis dose distributions for the symmetrically and asymmetrically focused 250 MeV VHEE are shown in Fig. 2, with unfocused 250 MeV VHEE for comparison. In both focusing cases the quadrupole strengths found by Elegant were meant to focus at 20 cm into the phantom; the resultant TOPAS simulations show that the actual position of the focal point in the water simulation varies due to scattering and the quadrupole set up used.

**Figure 3:**
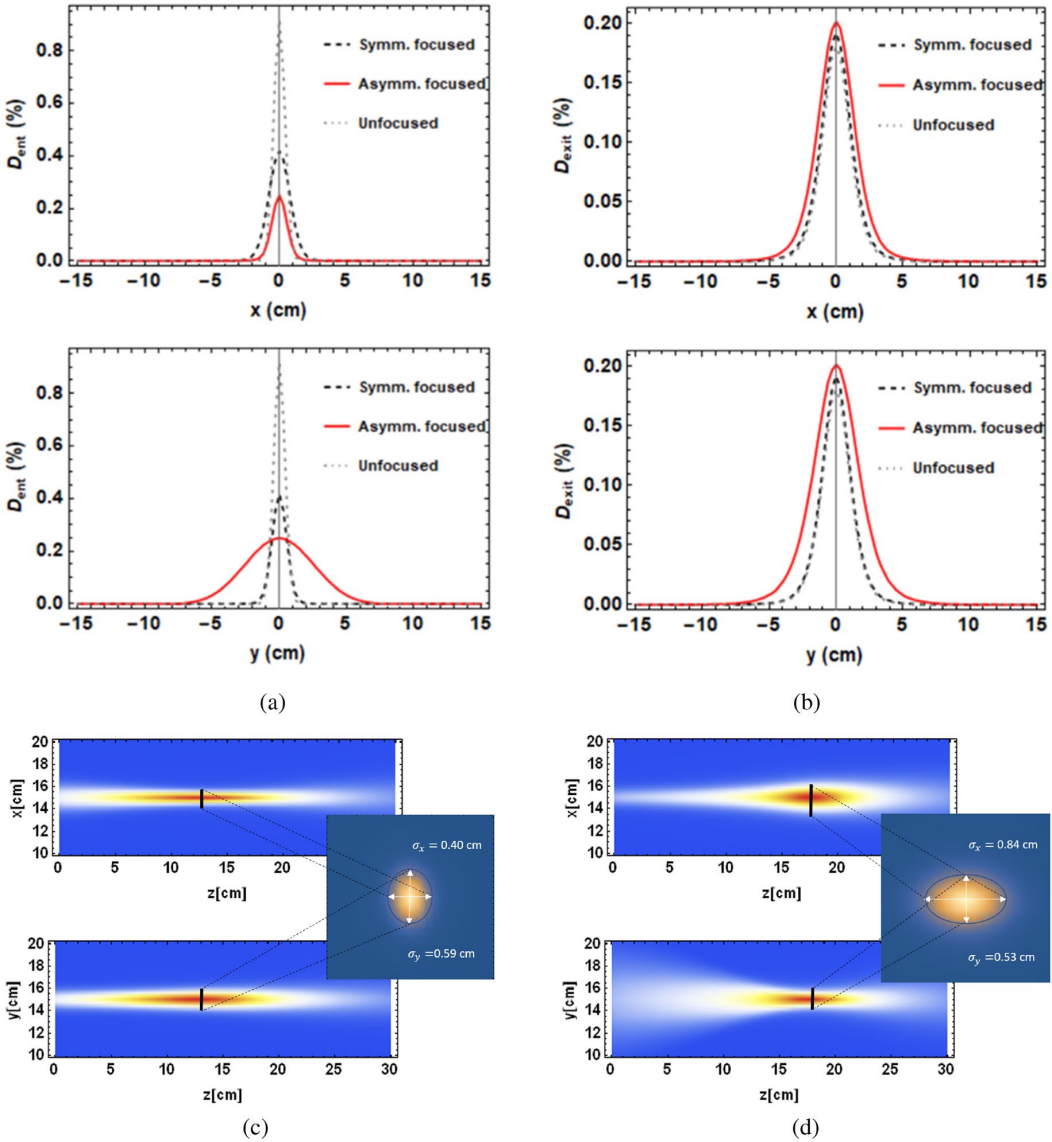
TOPAS-based Monte Carlo simulations of (**a**) the tranverse entrance and (**b**) transverse exit dose for 250 MeV symmetrically, asymmetrically and unfocused VHEE, with the dose normalised to the maximum dose for each case. The transverse dose maps for 250 MeV symmetrically and asymmetrically focused VHEE beams are shown in (**c**) and (**d**) respectively, with the dose cross sections at the focal point also shown.

The dose distributions in the transverse and longitudinal planes are shown in Fig. 3. These show that for approximately symmetrically focused beams that are less sharply focused in the on-axis dose distributions, the beam enters the phantom at a similar size in both transverse planes, and is not sharply focused at the focal point. For the asymmetrically focused beams, a stronger focusing effect occurs at the focal point but the effect is different in the x and y planes. This is due to the nature of the quadrupoles—each quadrupole produces focusing in one transverse plane whilst defocusing in the other transverse plane. The benefit to this type of focusing is that it spreads out the entrance dose over a wider area, resulting in a lower entrance dose at any point over the surface of the phantom, as shown in Fig. 3 and Table 1, whilst the exit doses remain similar for both focused and unfocused VHEE. The cross-sections of the dose at the focal point are also shown in Fig. 3. The corresponding Gaussian beam $\sigma _x$ and $\sigma _y$ at the focal point are shown in Table 1. For proton radiotherapy, the beam $\sigma$ at the Bragg peak is typically of the order of 10 mm at high energies ($\ge$100 MeV)^26^, meaning that the 250 MeV VHEE beam is smaller in both planes than a typical proton beam at the same depth. The Gaussian beam $\sigma _x$ and $\sigma _y$ at different depths through the water phantom is shown in Supplementary Fig. 1 in Supplementary Materials for symmetrically, asymmetrically and unfocused VHEE. This shows that the symmetric focusing has a minor effect on the shape of the dose distribution in the water phantom, whilst asymmetrically focused VHEE markedly changes this, with noticeable differences between the shape of the beam in the x- and y-planes.

**Table 1:** Beam $\sigma _x$ and $\sigma _y$ at focal point, depth of focal point ($\hat{z}$) and entrance doses ($D_{ent}$) for 250 MeV beams, each with initial Gaussian beam $\sigma =4$ mm.

Type of focusing	$\sigma _x$ (cm)	$\sigma _y$ (cm)	$\hat{z}$ (cm)	$D_{ent}$ (%)
No focusing	0.42	0.42	4	91.9
Symmetric beam	0.40	0.59	12.6	41.8
Asymmetric beam	0.84	0.53	17.4	25

While pencil-beam spot scanning using FLASH radiotherapy is under investigation, it is currently clear the FLASH effects are apparent for ultra-high dose rates and also very short treatment times (in many $<0.1$ s)^27^. For this reason, having a dose distribution that spreads out the entrance dose over a wide region and targets a volume in the patient could be a successful modality for FLASH VHEE radiotherapy. Therefore, due to the potentially beneficial nature of the dose distributions for asymmetrically focused VHEE, other beam energies were also investigated for the asymmetric case, with the aim of investigating the beam energies required for effective VHEE focusing to treat deep-seated tumours. The quadrupole strengths found for asymmetrically focused 250 MeV were adjusted for 100 MeV, 150 MeV and 200 MeV VHEE beams using the method shown in the “Methods” section, with the quadrupole positions remaining the same. The quadrupole strengths found are shown in Supplementary Table 2 in Supplementary Materials.

**Figure 4:**
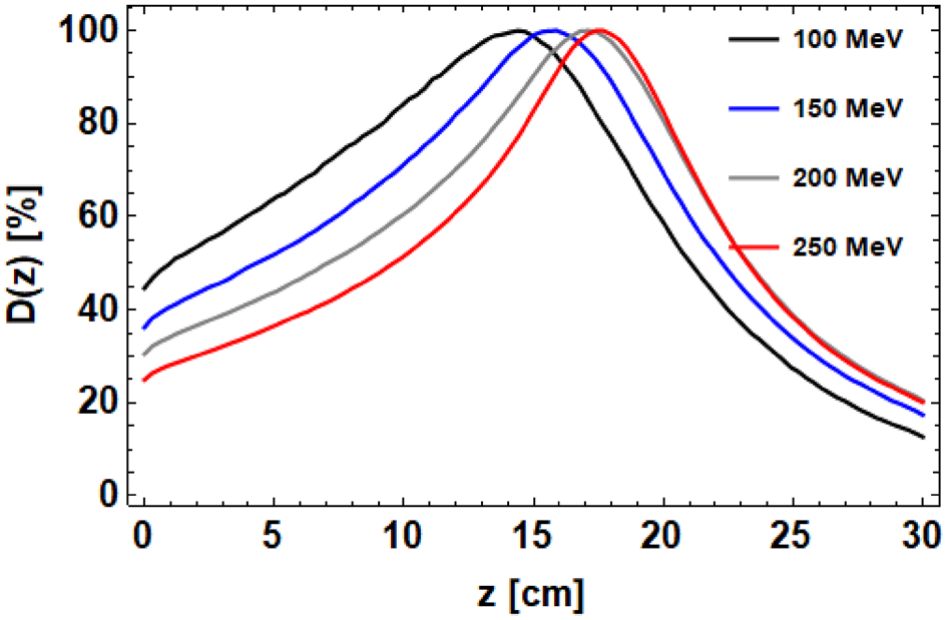
TOPAS-based Monte Carlo simulations of the on-axis dose plots for 100, 150, 200 and 250 MeV asymmetrically focused VHEE beams in water. Differences in focusing is due to the increasing penetration power and reduced scattering of VHEE with beam energy.

The on-axis dose distributions for the four different beam energies are shown in Fig. 4. The result is that higher energy beams require higher quadrupole strengths to focus them, and as such the on-axis dose distributions are more sharply focused for the higher energy VHEE than the lower energies. The entrance dose for the four beam energies are shown in Table 2, showing a reduction in dose by energy of 20% when increasing the beam energy from 100 to 250 MeV. This result also shows that the beam energy used changes the position of the maximum dose; this is due to the increasing penetration power and reduced scattering of VHEE with beam energy. The $\sigma _x$ and $\sigma _y$ of the dose distributions throughout the water phantom for the different beam energies are shown in Supplementary Fig. 1 in Supplementary Materials. This shows the differences in focusing in the x- and y-planes, as well as the reduced scattering with energy of the beam.

**Table 2:** Beam $\sigma _x$ and $\sigma _y$ at focal point, depth of focal point ($\hat{z}$) and entrance doses ($D_{ent}$) for 100, 150, 200 and 250 MeV asymmetrically focused beams in water.

Energy (MeV)	$\sigma _x$ (cm)	$\sigma _y$ (cm)	$\hat{z}$ (cm)	$D_{ent}$ (%)
100	1.84	1.15	14.4	45
150	1.30	0.80	15.9	36
200	1.02	0.65	17.1	31
250	0.84	0.54	17.4	25

**Figure 5:**
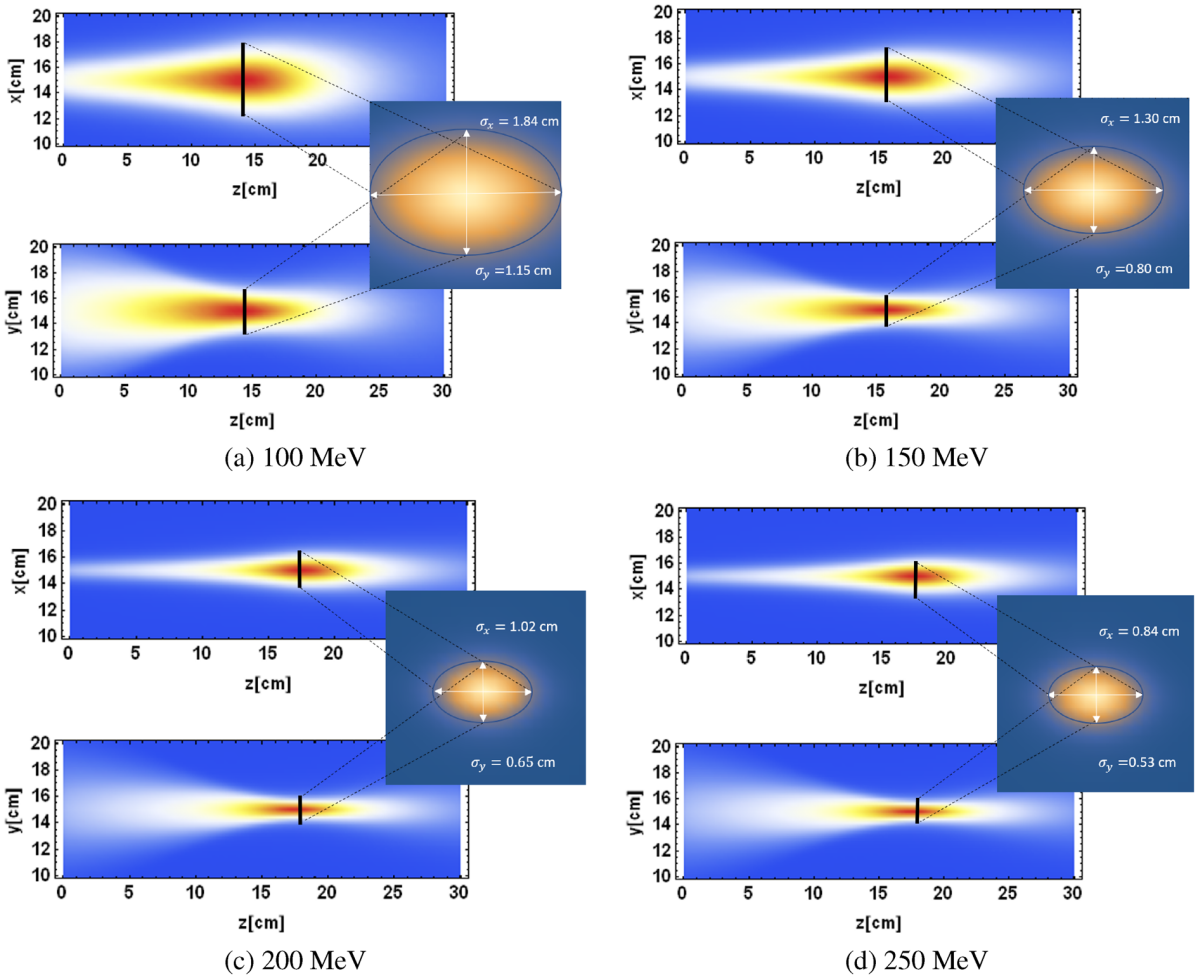
TOPAS-based Monte Carlo simulations of the transverse dose maps for 100 MeV, 150 MeV, 200 MeV and 250 MeV asymmetrically focused VHEE beams, with the dose cross sections at the focal points shown.

The dose distributions in the transverse and longitudinal planes for the four different beam energies are shown in Fig. 5. In all cases, the dose distribution is different in each transverse plane as expected, with the strongest focusing achieved for 250 MeV. The cross sections of the dose distributions at the focal point are also different for each beam energy, as shown in Fig. 5. It is clear that the higher energy beams produced smaller beam $\sigma$s at the focal point; this effect can be attributed to the reduced penumbral spreading with energy for VHEE, as well as the higher focusing strengths used.

The Gaussian $\sigma _x$ and $\sigma _y$ at the focal point for each of the four beam energies are shown in Table 2. This shows that by changing the beam energy from 100 to 250 MeV, the beam $\sigma$s at the focal point reduce by approximately 55%. Both the 200 MeV and 250 MeV VHEE have beam $\sigma$s approximately the same size or smaller as a typical 160 MeV proton Bragg peak in water^26^, with the 150 MeV beam $\sigma _x$ being only 3 mm larger. This implies that any of these beam energies could be clinically useful with regards to beam size at the target.

Scanning could be simplified if only one quadrupole was the variable, therefore the effect of changing the final quadrupole for each beam energy were investigated first analytically and then in terms of resultant dose distributions. The Twiss parameters^28^ (see “Methods” section) at the start of the phantom were found for each of the beam energies, and used to predict where the focal point should be. The resultant focal positions in the x- and y-planes were found for a range of different final magnet strengths for each of the beam energies. These were compared to the position of the maximum dose in the water phantom. The results are shown in Supplementary Table 3 in Supplementary Materials. It was found that the maximum of the dose distribution occurred at the same position in both the x- and y-planes, which was different to each of the focal positions predicted by Twiss parameter analysis. To investigate this, TOPAS simulations were run for 250 MeV VHEE with the same magnet parameters but in a vacuum, and again in air, in order to compare the results for each, shown in Supplementary Fig. 2 in Supplementary Materials.

It was found that there was a difference of 1–2 cm in the position of the focused dose between the analytical predictions from the Twiss parameters from Elegant^24^ and the full water phantom simulation. This is likely due to scattering of the beam in the water phantom producing a maximum in the dose at a depth less than that in a vacuum. A small difference in the results for the analytical prediction and the vacuum results is likely due to space charge effects or differences in the code of how the quadrupole magnets are simulated. The TOPAS simulations though, through many PDD simulations compared to experiments, have been show to be robust and accurate^29^.

**Figure 6:**
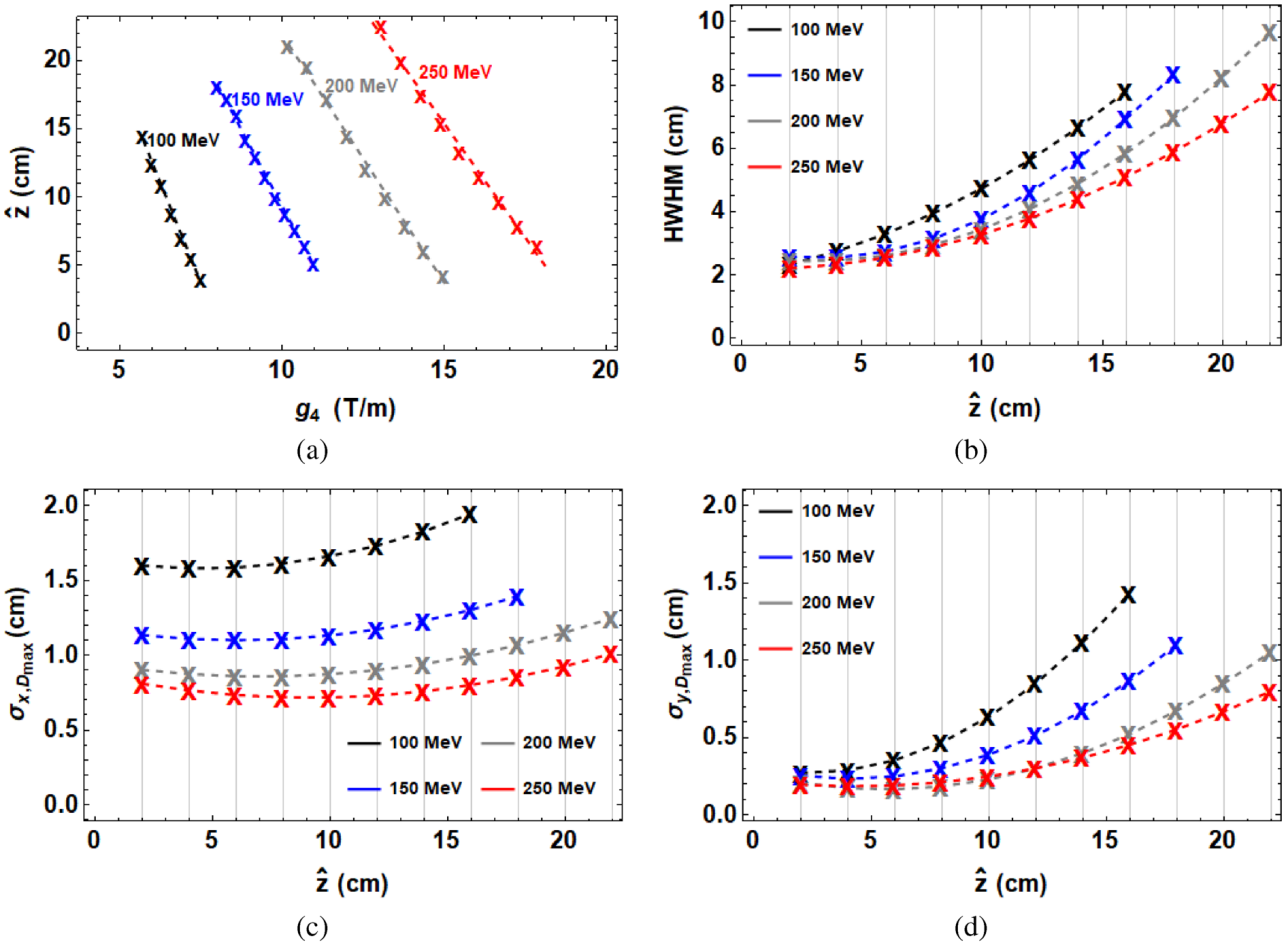
TOPAS-based Monte Carlo simulations of the (**a**) Position of the maximum dose (i.e. the focal point) as a function of the final quadrupole strength for the set up shown in Supplementary Table 2 in Supplementary Materials together with a fit based on Eq. (1). (**b**) *HWHM* of the on-axis dose distribution as a function of focal dose position, $\hat{z}$ together with a similar fit to that in Eq. (2) also shown. Shown in (**c**) and (**d**) are TOPAS-based simulations for $\sigma _x$ and $\sigma _y$ at the focal point, together with quadratic fits to them based on Eq. (2). All simulations track $10^7$ particles within a water phantom.

Changing the strength of the final quadrupole has an effect on the width and position of the resultant on-axis dose distribution, as well as the shape of the dose distribution in the transverse planes. Figure 6 shows how the position of the maximum dose (i.e. the focal point) changes by changing the final quadrupole strength for each of the 4 beam energies. This could be used to cover a tumour with any length inside the patient, just by changing the final quadrupole strength. The relationship between the position of maximum dose (i.e. the focal point), $\hat{z}$ (m) and final quadrupole strength, $g_4$ (T/m) was found to be approximately linear. Accounting for the beam energy, *E* (MeV) as well, this fit is extended by including quadratic terms, with the general formula

1 $$\begin{aligned} \hat{z}(g_4,E) = a_1 g_4^2 + a_2 g_4 + a_3 E^2 + a_4 E + a_5 \end{aligned}$$

where $a_1$, $a_2$, $a_3$, $a_4$ and $a_5$ are constants obtained from a least square error fit. This readily enables us to predict intermediate energy behaviour. The fits for position of maximum dose as a function of final magnet strength and energy for the plots shown in Fig. 6 are given in Table 3. The $R^2$ value for the goodness of fit is 0.999. It is straightforward to convert $\hat{z}$ to cm as all that is required is to multiply by 100.

**Table 3:** Fit parameters and associated errors for Eq. (1).

Parameter	Value
100 $a_1$ [m$^3$T$^{-2}$]	$0.11 \pm 9.7\times {10^{-3}}$
100 $a_2$ [m$^2$T$^{-1}$]	$-$ $6.63 \pm 2.6 \times {10^{-3}}$
100 $a_3$ [m(MeV)$^{-2}]$	$-$ $5.16 \times {10^{-4}} \pm 4.21\times {10^{-5}}$
100 $a_4$ [m(MeV)$^{-1}]$	$0.46 \pm 0.017$
100 $a_5$ [m]	$7.16 \pm 0.80$

Changing the final quadrupole strength also affects the shape of the beam. Knowledge of how the beam shape changes with quadrupole strength could be used to shape the beam to the tumour at a particular location. The relationship between the beam $\sigma _{x}$ in m at the focal point as a function of the final quadrupole strength, $g_4$ (T/m) was found to be approximately quadratic, with the general formula

2 $$\begin{aligned} \sigma _x(g_4) = a_1 g_4^2 + a_2 g_4 + a_3, \end{aligned}$$

where $a_1$, $a_2$ and $a_3$ are constants obtained from a least square error fit, and with a similar relation for $\sigma _y$. The fits for the beam $\sigma _x$ and $\sigma _y$ at the focal point as a function of the final quadrupole strength for the four beam energies are given in Table 4. For $\sigma _x$ and $\sigma _y$ in cm we simply multiply by 100.

**Table 4:** Fit parameters and associated errors for Eq. (2) for a range of energies, E.

E (MeV)	Fitted $\sigma _{x,y}$ (cm)	100 $a_1$ (m$^3$T$^{-2}$)	100 $a_2$ (m$^2$T$^{-1}$)	100 $a_3$ (m)
100	$\sigma _x$	$0.10 \pm 8.61\times {10^{-3}}$	$-$ $1.41 \pm 0.11$	$6.76 \pm 0.37$
100	$\sigma _y$	$0.21 \pm 0.01$	$-$ $3.31 \pm 0.16$	$13.00 \pm 0.53$
150	$\sigma _x$	$0.04 \pm 3.19\times {10^{-3}}$	$-$ $0.87 \pm 0.06$	$5.78 \pm 0.29$
150	$\sigma _y$	$0.09 \pm 5.51\times {10^{-3}}$	$-$ $1.98 \pm 0.11$	$11.30 \pm 0.49$
200	$\sigma _x$	$0.02 \pm 2.84\times {10^{-3}}$	$-$ $0.65 \pm 0.07$	$5.44 \pm 0.44$
200	$\sigma _y$	$0.04 \pm 3.77\times {10^{-3}}$	$-$ $1.30 \pm 0.10$	$9.74 \pm 0.59$
250	$\sigma _x$	$0.02 \pm 1.32\times {10^{-3}}$	$-$ $0.67 \pm 0.04$	$6.40 \pm 0.32$
250	$\sigma _y$	$0.02 \pm 7.89\times {10^{-4}}$	$-$ $0.80 \pm 0.25$	$7.54 \pm 0.19$

**Figure 7:**
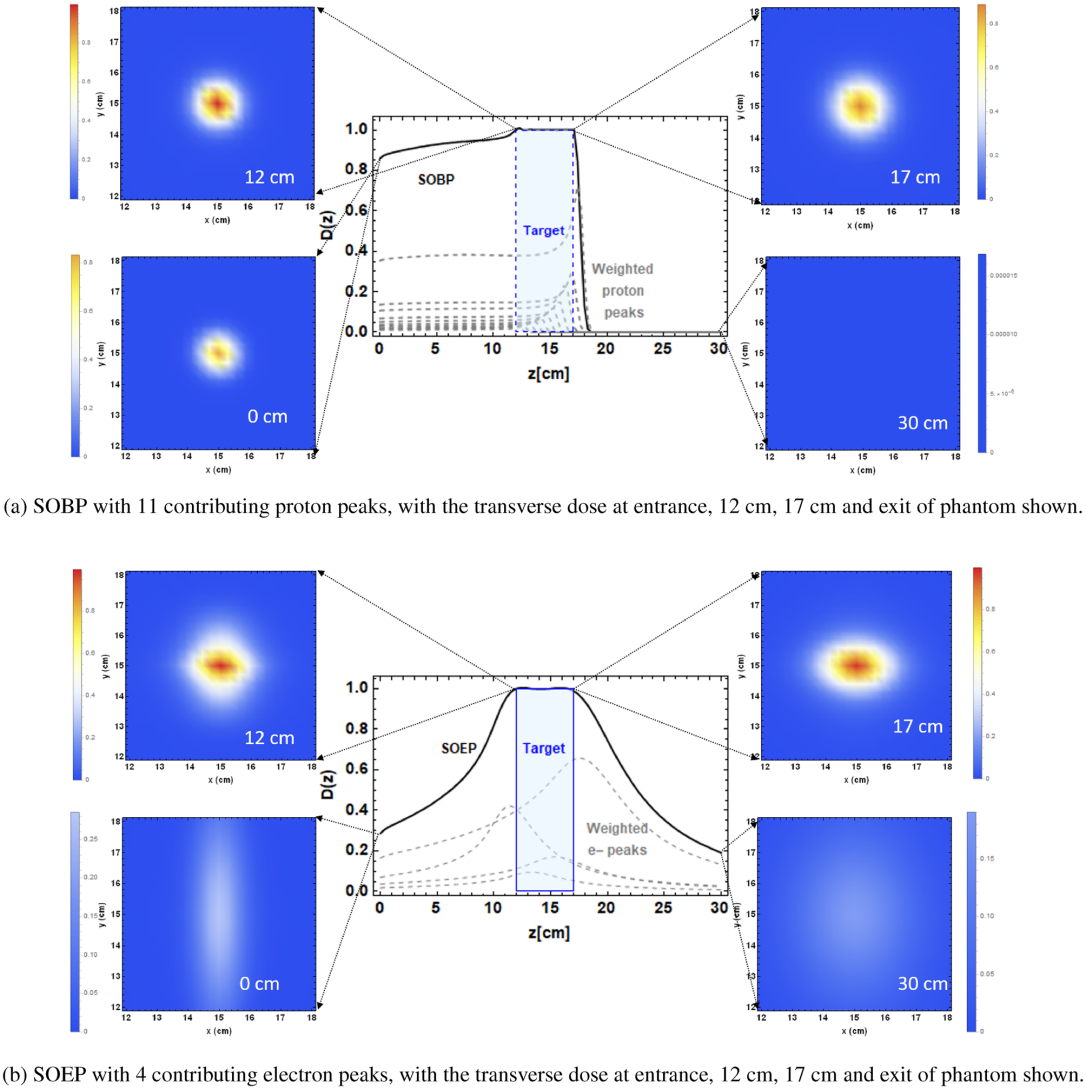
TOPAS-based Monte Carlo simulations of the spread-out electron peak produced using 250 MeV VHEE, the quadrupole set up shown in Supplementary Table 2 and optimisation based on Eq. (16), changing only the final quadrupole strength $g_4$ to shift the peak position. The corresponding spread-out Bragg peak (SOBP) over the same region is also shown, both with the target located 12–17 cm into the water phantom. In both cases, the transverse dose cross sections are shown at the entrance, beginning of the target, end of the target and exit.

The fits produced for position of focal point as a function of final magnet strength, $g_4$ and $\sigma _{x,y}$ as a function of $g_4$ were used to produce a fit of $\sigma _{x,y}$ as a function of focal point position, $\hat{z}$. The results of these fits are shown in Fig. 6c, d. These results could be used to predict the beam size for a required depth of maximum dose. The half-width-half-maxima (HWHM) of the on-axis dose distributions for each of the beam energies and magnet strengths are shown in Fig. 6. This shows that focusing is most efficient close to the surface, due to the higher focusing strengths used and reduced beam scattering. As a first step towards showing how focused VHEE could be used to cover a tumour region, a spread-out electron peak (SOEP) has been produced for 250 MeV VHEE, shown in Fig. 7 by changing the final quadrupole strength. Mathematica^30^ was used to optimise for flatness over a region of 12–17 cm into the water phantom, i.e. corresponding to a target size of 5 cm. This was done by taking the on-axis dose for each of the different magnet strengths and multiplying by a variable weighting factor, then minimising the deviation from unity by summing the weighted doses over the region by changing each of the weights. This method mimics how multiple proton beams are combined using weighting factors to produce a spread-out Bragg peak (SOBP), with the idea that multiple SOEPs or SOBPs could be combined to treat a 3D tumour region. The resultant on-axis SOEP and SOBP covering the target region are shown in Fig. 7, with parameters shown in Table 5. The transverse dose distributions at the entrance, start of the target, end of the target and exit of the phantom are also shown in Fig. 7, showing the shape and intensity of the SOEP dose and SOBP dose through the water phantom. The colour of the dose distributions are all normalised to the maximum dose in the SOEP or SOBP. This shows the difference between the two types of beams; the proton SOBP has a higher entrance dose, but no exit dose and the dose is contained within approximately 1 cm transversely. The SOEP has a lower entrance dose, but the dose is spread out over a wider region at the entrance, and there is some exit dose. In both cases the target region is covered. The transverse dose distributions of the SOEP and SOBP throughout the water phantom in increments of 3 cm are shown in Supplementary Figs. 4 and 5 Supplementary Materials.

**Table 5:** Spread-out electron peak (SOEP) and spread-out Bragg peak (SOBP) characteristics.

Parameters	SOEP	SOBP
Beam energy (MeV)	250	130–160
Entrance dose ($\%$)	28.6	85.9
Exit dose ($\%$)	19.0	0
Flatness ($\%$)	99.5	99.6
Number of peaks	4	11

## Discussion

Focusing of deeply penetrating VHEE beams using realistic quadrupole magnets in Monte Carlo simulations has been shown here. The on-axis dose distribution and transverse dose distributions show a significant reduction in entrance dose and similar exit dose compared to unfocused VHEE, and the use of the quadrupole magnets means the position of the maximum dose can be chosen by simply changing the quadrupole strengths. The reduction in entrance dose is highest for 250 MeV VHEE focused asymmetrically, although the reduction in entrance dose is only 20% from 100 to 250 MeV, compared to 47% for unfocused 250 MeV VHEE and 100 MeV focused VHEE. The quadrupole magnets shape both the on-axis dose distributions and the shape of the beam in both transverse planes. By careful selection of the magnet strengths, the shape of the beam at the focal point can be pre-selected with varying beam $\sigma$s in the x-plane and y-plane. The effect of changing the beam energy from 100 to 250 MeV results in the beam $\sigma$s at the focal point being reduced by approximately 55%, from $\sigma _x=1.84$ cm and $\sigma _y=1.15$ cm for 100 MeV to $\sigma _x=0.84$ cm and $\sigma _y=0.53$ cm for 250 MeV. For the 150 MeV, 200 MeV and 250 MeV VHEE, the beam $\sigma$s at the focal point are all approximately the same order of magnitude as the corresponding proton Bragg peak in air at the same depth (15 cm). This implies any of these beam energies could be appropriate for focused VHEE radiotherapy.

Previous Monte Carlo studies in focused VHEE radiotherapy have mostly involved theoretical focusing by pre-selecting focusing strengths in the Monte Carlo simulation^31^ with no attachment to realistic magnetic properties. This study goes a step further towards realistic focusing of VHEE radiotherapy by investigating the focusing strengths and number of realistic quadrupole magnets required to produce focusing in both transverse planes in a semi-analytical way, in contrast to previous studies in proton focusing (see for instance^21^)which take a simple FODO lattice using available magnets, rather than optimised magnet parameters. Two experimental studies^22,23^ have been completed so far focusing VHEE beams at the CLEAR facility, with focal depths of approximately 5 cm into the water phantom and focusing in only one plane. This study shows for the first time that by changing the strength of only the final quadrupole magnet, the position of the maximum dose can be changed, enabling dose coverage of a whole region (> 20 cm) by changing the final quadrupole strength. This relationship is linear over the region of the water phantom for each of the four beam energies, so this could potentially be done in a continuous manner, which could be useful in a clinical setting when dose must be delivered in a short time frame. Predicting the position of the focal point from beam energy and final quadrupole strength is possible by extending this fit to include quadratic terms. This study shows for the first time that combining multiple focused VHEE beams produced simply by changing the final quadrupole strength allows for a spread-out electron peak (SOEP) to be produced over a target region on-axis, with a lower entrance dose than the corresponding on-axis spread-out Bragg peak (SOBP), a low exit dose, and with a similar flatness to the SOBP. This is a stepping stone towards 3D focused VHEE treatment planning, with the idea that multiple SOEPs could be combined to create a 3D target dose distribution.

The results in this study were produced by using four quadrupoles for asymmetrically focused beams and six quadrupoles for the symmetrically focused beams; this is not optimal for a clinical setting. In addition the quadrupole magnets are placed very close to the phantom and the beam size within the quadrupoles is not constrained.

Generally, clinically useful beams have been produced in Monte Carlo simulations in this study, and the magnet parameters required for an experiment to show realistic VHEE beam focusing have been found. Such an experiment would be possible at the CLEAR facility at CERN, with minimal changes required to account for the achievable beam parameters. This experiment would validate the transverse and longitudinal dose distributions of focused VHEE beams, the production of a spread-out electron peak by changing only the final quadrupole strength, as well as the potential for continuous scanning of the final quadrupole strength to cover a range of depths in a water phantom. Future research is required to translate this into a clinical setting, in particular how to reduce the size of the apparatus, and the possibility of combining the set-up with a dipole magnet steering system to produce a VHEE treatment planning system.

## Conclusions

Monte Carlo simulations performed in this study show that it is possible to focus very high energy electron (VHEE) beams to produce high dose volumes inside a water phantom at depths required for deep-seated tumours, and that it is possible to change the shape of the dose distribution by changing only the quadrupole configuration. Symmetric and asymmetric focusing was investigated for 250 MeV VHEE, with lower entrance dose for the asymmetric case. The effect of beam energy on focusing was investigated for 100 MeV, 150 MeV, 200 MeV and 250 MeV asymmetrically focused VHEE, with the beam $\sigma$s all approximately 1 cm at the focal point for 150 MeV, 200 MeV and 250 MeV VHEE. Increasing the beam energy from 100 to 250 MeV decreased the entrance dose by a further 20%, from 45 to 25%. The on-axis dose distributions show reduced entrance doses and similar exit doses compared with unfocused VHEE beams. This study also shows that the position of the maximum dose can be changed by changing only the final quadrupole strength for each of the four beam energies, allowing for the possibility of producing spread-out electron peaks (SOEPs) similar to spread-out Bragg peaks (SOBPs) simply by altering the final magnet strength.The relationship between maximum dose position and final quadrupole strength is approximately linear, allowing for this to be performed potentially in a continuous manner during dose delivery. The position of the maximum dose can also be predicted by a straightforward two parameter quadratic fit involving beam energy and final quadrupole strength. Further research is required into how to translate focused VHEE into a clinical setting, including reducing the number of quadrupole magnets required and combining the magnets with a VHEE treatment planning system.

## Methods

### Elegant and Monte Carlo simulation details

**Figure 8:**
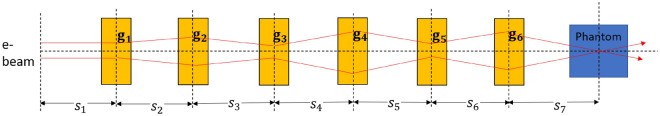
Schematic of the quadrupole set-up used in the symmetric focusing Monte Carlo simulation. The same set up sans the final two quadrupoles was used for all asymmetrically focused VHEE simulations. For the PDD curves and unfocused VHEE simulations, only the beam, drift space of 5 cm air and the water phantom were simulated.

The Elegant code was first used to optimise for the number, position and strengths of 18 cm long quadrupole magnets to focus a 4 mm $\sigma$ Gaussian starting beam at 20 cm in a phantom. This involved multiple iterations and the code works in a vacuum with no space charge effects. A lattice was modelled as consisting of six quadrupole magnets and eight drift spaces, with the final drift space corresponding to the focusing depth into the phantom. Elegant optimised the magnet strengths and drift spaces required for minimum $\alpha$ and $\beta$ at a depth of 20 cm, with the constraints that the drift spaces could not be more than 2 m, and quadrupole strengths could not be over 27 T/m. The magnet parameters found are shown in Supplementary Tables 1 and 2 in Supplementary Materials. For the TOPAS Monte Carlo simulations, the symmetric, antisymmetric and unfocused VHEE beams were each a 4 mm initial $\sigma$ Gaussian 250 MeV VHEE beam with 0.75 MeV energy spread, and 3.2 mrad angular divergence, parameters common for clinical proton beams^32^. For the comparison of the different beam energies, VHEE beams of energy 100 MeV, 150 MeV, 200 and 250 MeV were used, each with initial Gaussian $\sigma$s of 4 mm, 0.75 MeV energy spread and 3.2 mrad angular divergence. The quadrupoles were simulated to consist of air, and of size 40 cm $\times$ 40 cm $\times$ 18 cm. The water phantom is 30 cm $\times$ 30 cm $\times$ 30 cm. The set-up simulated in TOPAS is shown in Fig. 8. The dose deposited in the water phantom was scored using TOPAS in voxels of 101 $\times$ 101 $\times$ 101 voxels to produce comma separated variable (CSV) files. For each of the proton beam simulations, a 0.75 MeV energy spread, 3.2 mrad angular divergence and 4 mm initial $\sigma$ Gaussian beam were used^32^. The proton energy of the beam shown in Fig. 1 was 150 MeV. The proton energies for the SOBP are shown in the SOBP section. The photon beam was modelled as a 2.5 MV energy photon beam with 0.0075 MV energy spread, 3.2 mrad angular divergence and 4 mm initial $\sigma$. The 15 MeV electron beam was simulated as a 15 MeV 4 mm initial $\sigma$ electron beam with 0.075 MeV energy spread and 3.2 mrad angular divergence. The 320 MeV/n carbon-12 ion beam was simulated as a 3840 MeV 4 mm initial $\sigma$ Gaussian carbon-ion beam with 0.384 MeV energy spread (0.01%) and 3.2 mrad angular divergence. In each case, $10^7$ particles were used.

### Data processing and analysis

The data was analysed in Mathematica^30^ to produce plots and determine beam sizes and dose distributions at various points within the water phantom.

### Dose distributions

The integrated dose distributions were produced by scoring the dose deposited in 1 $\times$ 1 $\times$ 101 voxels through the water phantom for each of the different particle types. The on-axis dose distributions were produced by taking the dose deposited in the middle voxel in the x and y planes (voxel 50), i.e. the dose deposited in a 0.297 cm $\times$ 0.297 cm $\times$ 30 cm slice through the centre of the water phantom. The cross-sectional dose at the focal length for each focused beam was produced by plotting the dose deposited in a 30 cm $\times$ 30 cm $\times$ 0.297 cm slice through the water phantom at the focal point (position of maximum dose). Mathematica^30^was used to fit Gaussian distributions to the dose distributions to determine the beam $\sigma$ in the x- and y-planes at the focal point. For the beam $\sigma$ at the entrance of the phantom, Mathematica^30^ was used to fit Gaussian distributions to the dose deposited in a 30 cm $\times$ 30 cm $\times$ 0.297 cm slice at the start of the water phantom (i.e. voxel 0). The transverse dose maps were produced by taking a 30 cm $\times$ 0.297 cm $\times$ 30 cm slice for the x–z dose map through the centre of the y-plane (i.e. voxel 50), and by taking a 0.297 cm $\times$ 30 cm $\times$ 30 cm slice for the y–z dose map through the centre of the x-plane (i.e. voxel 50).

### Evaluation of beam focusing position using Twiss parameters

Particle motion in an accelerator is described by the Hill’s equation^28^,

3 $$\begin{aligned} \frac{d^2 x}{ds^2} + K(s) x = 0 \end{aligned}$$

where *x* is the particle’s displacement from the central orbit, *s*, and $x^{\prime } =\frac{dx}{ds}$ is the angle with respect to the central orbit, and *K*(*s*) is the focusing strength at a particular point along the central orbit (in practice *K* is piecewise linear). The general solutions to Hill’s equation are

4 $$\begin{aligned} x(s)= & {} \sqrt{\epsilon \beta (s)} \cos {\psi (s)} \nonumber \\ x^{\prime }(s)= & {} -\alpha \sqrt{\epsilon / \beta (s)} \cos {\psi (s)}- \sqrt{\epsilon / \beta (s)} \sin {\psi (s)} \end{aligned}$$

where $\epsilon$ is the beam emittance, $\beta (s)$ is the amplitude modulation due to changing focusing strength which combined with $\alpha$ and $\gamma$ $\left( =\frac{1+\alpha ^2(s)}{\beta (s)}\right)$ make up the Twiss parameters. Also, $\psi (s)$ is the phase advance. All particles in the beam travel along their own trajectories within an ellipse in phase space $(x,x')$ which can be shown^28^ to be of area

5 $$\begin{aligned} \int _{\text {ellipse}}\text {d} x(s) \text {d} x^{\prime }(s) =\pi \epsilon = \pi \left( \gamma (s) x^2(s) + 2 \alpha (s) x(s) x^{\prime }(s) + \beta (s) x^{\prime 2}(s) \right) \end{aligned}$$

**Figure 9:**
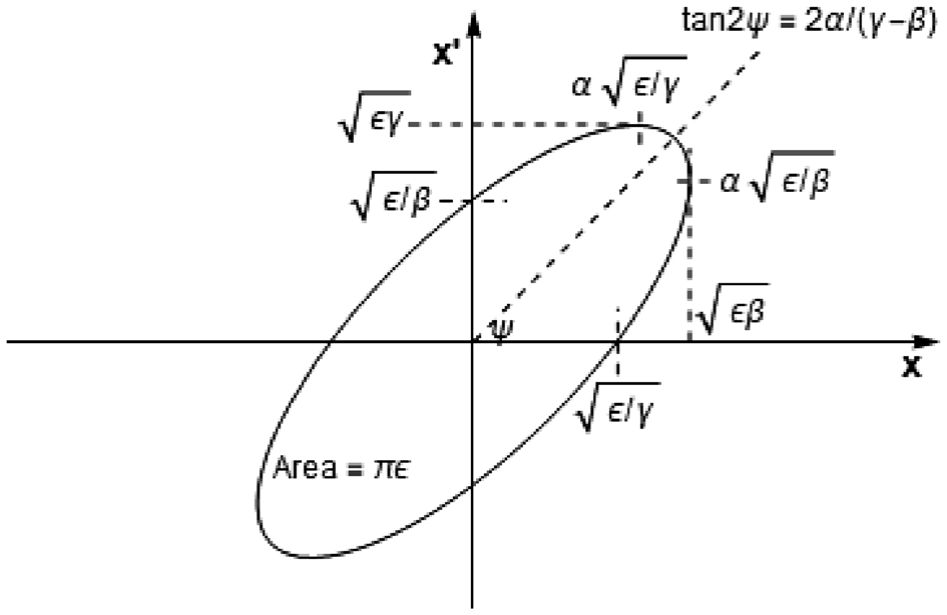
Parameters associated with an ellipse in phase space.

Choosing the particle on the largest phase ellipse within a beam means that all the particles will stay within that ellipse, allowing for behaviour of the whole beam to be described by a single particle. This is shown in Fig. 9.

The particle’s transverse position and angle vary throughout the machine due to differences in focusing strength, and can be represented by a matrix. The modulations due to different types of magnets can be represented as a Transport Matrix, *M*, between two points, $(x_i,x^{\prime }_i)$ and $(x_f,x^{\prime }_f)$

6 $$\begin{aligned} \begin{pmatrix} x_f\\ x^{\prime }_f \end{pmatrix} = M(x_f,x_i) \begin{pmatrix} x_i\\ x^{\prime }_i \end{pmatrix}. \end{aligned}$$

As in the literature^33^, the matrix element for a focusing quadrupole $M_{QF}$ of length $L_Q$ and focusing strength *K* is given by similarly

7 $$\begin{aligned} M_{QF}=\begin{pmatrix} \cos {\Theta } &{} \frac{1}{\sqrt{|K |}} \sin {\Theta }\\ -\sqrt{|K |} \sin {\Theta } &{} \cos {\Theta } \end{pmatrix} \end{aligned}$$

where $\Theta = \sqrt{|K |}L_Q$. The matrix element for a defocusing quadrupole, $M_{QD}$ of length $L_Q$ and strength $-K$ is similarly given by

8 $$\begin{aligned} M_{QD}=\begin{pmatrix} \cosh {\Theta } &{} \frac{1}{\sqrt{|K |}} \sinh {\Theta }\\ \sqrt{|K |} \sinh {\Theta } &{} \cosh {\Theta } \end{pmatrix}. \end{aligned}$$

Note that the magnetic field gradient, *g* (T/m) of a quadrupole magnet can be found from the focusing strength, *K* (m$^{-2}$), via

9 $$\begin{aligned} g \approx EK/300 \end{aligned}$$

where *E* is the beam energy (MeV). Note here the speed of light has been approximated as $3\times {10^8}$ m/s and the beam travelling with velocity *v* is assumed to be sufficiently relativistic that $\beta$ = $v/c \approx 1$. For a drift space, the matrix element, $M_{d}$ for a drift of length *L* (m) is given by

10 $$\begin{aligned} M_d = \begin{pmatrix} 1 &{} L \\ 0 &{} 1 \end{pmatrix}. \end{aligned}$$

The transfer matrix for the full four quadrupole setup can be found by multiplying the matrices,

11 $$\begin{aligned} M = M_{d_5} M_{QD_2} M_{d_4} M_{QF_2} M_{d_3} M_{QD_1} M_{d_2} M_{QF_1} M_{d_1} \end{aligned}$$

with a similar transfer matrix for the six quadrupole case.

The Twiss parameters $\alpha , \beta , \gamma$ $(=(1+\alpha ^2)/\beta )$ characterise the beam electron trajectories. They are related to the magnet strengths and drift spaces the particles traverse. Our aim is to focus the beam at a particular depth, *L*, within the phantom. Equation (7) allows us to evaluate the beam position ($x,x'$) in phase space after progressing through all of the quadrupole magnets and a water phantom—all encompassed within the matrix *M*. We assume the water phantom is modelled by a drift space of length $L_p$(no scattering included) and we can now propagate the Twiss parameters through all of the magnets using Eq. (12) and obtain their values at the phantom by

12 $$\begin{aligned} \epsilon \begin{pmatrix} \beta _f &{} - \alpha _f \\ - \alpha _f &{} \gamma _f \end{pmatrix} =M \epsilon \begin{pmatrix} \beta _i &{} - \alpha _i \\ - \alpha _i &{} \gamma _i \end{pmatrix} M^T \end{aligned}$$

where the emittance variation is assumed to be negligible. Doing separate calculations for the Twiss parameters from the start of the beam to the beginning of the phantom, and then reusing Eq. (13) with the new Twiss parameters ($\alpha _i, \beta _i$) and $M=M_d$ for the phantom of focal length L, here we can readily find

13 $$\begin{aligned} \alpha _f = \alpha _i - \frac{(1+\alpha _i^2)L}{\beta _i}. \end{aligned}$$

Now at the focal point, $\alpha _f=0$ and thus we finally arrive at the important relation between the focusing depth and the Twiss parameters

14 $$\begin{aligned} L=\frac{\alpha _i \beta _i}{1+\alpha _i^2} = \frac{\alpha _i}{\gamma _i}. \end{aligned}$$

The $\alpha _i$ and $\gamma _i$ are properties of the combined magnet configurations and we obtain them by running the Elegant code for specific magnet strengths and separations. Once we have these Twiss parameters, we can readily, through Eq. (15), predict the focal point. This is then used as a starting point in the TOPAS simulations (which include all relevant scattering parameters present in water and air). This has been independently produced by Elegant simulations, TOPAS simulations and Mathematica calculations, correct to within 5 mm.

### Changing magnet strength

The effect of changing the final magnet strength was investigated for 100 MeV, 150 MeV, 200 MeV and 250 MeV asymmetrically focused beams. TOPAS simulations were performed using the parameters found for each of the beam energies, changing the final quadrupole strengths in increments of 0.3–0.6 T/m until a range of approximately 15–20 cm into the water phantom was covered. The position of the peak depth for each of the beams produced was determined using Mathematica. The half-width-half-maxima (HWHM) of the on-axis dose distributions were determined by taking the difference of the position of the 0.5 $\times$ maximum dose and the position of the maximum dose. The $\sigma _{x,y}$ at the focal point were determined by fitting Gaussian distributions to the dose distribution deposited in the 30 cm $\times$ 30 cm $\times$ 0.297 cm slice at the focal point.

The positions of the focal points from the Twiss parameters for the 250 MeV beam were found using the Elegant code for the different focusing strengths shown in Supplementary Table 1 (asymmetric beam) in Supplementary Materials to produce the Twiss parameters at the entrance to the phantom, and then using the equation shown in the Twiss parameter analysis section, the corresponding focal lengths in the water phantom were found for each of the x- and y-planes. The focal lengths in the x-plane for each of the different final magnet strengths were then plotted with the positions of the maxima of the dose distributions from TOPAS^25^. These were found by running the simulation as described in the Monte Carlo simulation details section, but with the whole simulation in only a vacuum for the vacuum case, and only air for the air case, each with beam energy of 250 MeV and the focusing strengths shown in Supplementary Table 1 (asymmetric beam) in Supplementary Materials. 501 $\times$ 501 $\times$ 501 voxels were used here and the central voxel was taken to find the on-axis dose distribution to increase the precision of the maximum dose position.

### SOEP

The spread-out electron peak corresponds to delivering a flat distribution of dose over the target region. We were able to achieve this by summing a series of *n* weighted electron dose profiles with various magnet strengths. Here we only needed to vary the final quadrupole magnet strength, $g_4$. Over the target region, we minimised the following equation

15 $$\begin{aligned} f(a_1,a_2,\ldots ,a_n)= \int ^{z_{max}}_{z_{min}}\sum ^n_{i=1} (a_i D_i(z)-1)^2 dz \end{aligned}$$

where $D_i(z)$ corresponds to the dose profile for a particular magnet strength, $a_i$ to a weighting factor (varied), and $z_{min,max}$ to the location of the minimum and maximum of the target region. We used the Mathematica code^30^ to perform this minimisation. We characterised the flatness over the target region with

16 $$\begin{aligned} \delta = \left( 1-\frac{\sigma _D}{\langle D\rangle }\right) \times 100 \end{aligned}$$

where $\sigma _D$ is the standard deviation of the dose and $\langle D\rangle$ is the mean dose. For the 5 cm target, at a depth of 12–17 cm, we found no more than four focused beams were necessary to produce an on-axis field flatness of better than 99%. It is worth noting that a similar optimised SOEP can be achieved by varying the energy (rather than the magnet strength). Indeed, for protons we obtained a spread-out Bragg peak (SOBP) by varying the energy—but in this case we needed more than twice as many separate energies. The transverse dose at various depths through the SOEP and SOBP were produced by summing the weighted doses of each of the contributing beams and plotting the dose deposited in a 30 cm $\times$ 30 cm $\times$ 0.297 cm slice through the water phantom at depths from 0 to 30 cm in increments of 3 cm, as well as the dose at 17 cm (i.e. the edge of the target region).

## Supplementary Information

Supplementary Information 1.

## Data Availability

The datasets generated during and/or analysed during the current study are available from the corresponding author on reasonable request.
