# Supplementary material for: Focused VHEE (very high energy electron) beams and dose delivery for radiotherapy applications
Source: Sci Rep. 2021 Jul 7;11:14013. doi: 10.1038/s41598-021-93276-8 (PMC8263594; doi:10.1038/s41598-021-93276-8)
Supplement: Supplementary file 1 — Supplementary Information 1. [file 41598_2021_93276_MOESM1_ESM.pdf]

Focused VHEE (Very High Energy Electron) Beams and Dose Delivery For Radiotherapy Applications

Supplementary Information

Supplementary Table 1 shows the quadrupole strengths and positions obtained by the Elegant particle physics code to focus 250 MeV VHEE beams symmetrically and asymmetrically.

Supplementary Table 1. Quadrupole strengths and positions required to focus the 250 MeV beams.

Lattice parameter	Symmetric	Asymmetric
g_1 [T/m]	9.3	-10.5
s_1 [cm]	109	109
g_2 [T/m]	-17.5	10.6
s_2 [cm]	34	97.9
g_3 [T/m]	26.7	-8.0
s_3 [cm]	37.9	120.9
g_4 [T/m]	-5.5	14.3
s_4 [cm]	76.6	88.3
g_5 [T/m]	20.9	–
s_5 [cm]	31.74	–
g_6 [T/m]	-24.0	–
s_6 [cm]	28.9	–
s_7 [cm]	38.2	46.1

Supplementary Table 2 shows the quadrupole strengths and positions obtained by the Elegant particle physics code to focus 100 MeV, 150 Mev, 200 MeV and 250 MeV asymmetrically. The only change for each beam energy is the scaling of the quadrupole strengths; the quadrupole positions remain the same for each case.

Supplementary Table 2. Quadrupole strengths and positions required to focus the 100, 150, 200 and 250 MeV beams.

Lattice parameter	100 MeV	150 MeV	200 MeV	250 MeV
g_1 [T/m]	-4.2	-6.3	-8.4	-10.5
s_1 [cm]	109	109	109	109
g_2 [T/m]	4.2	6.3	8.5	10.6
s_2 [cm]	97.9	97.9	97.9	97.9
g_3 [T/m]	-3.2	-4.8	-6.4	-8.0
s_3 [cm]	120.9	120.9	120.9	120.9
g_4 [T/m]	5.7	8.6	11.4	14.3
s_4 [cm]	88.3	88.3	88.3	88.3
s_5 [cm]	46.1	46.1	46.1	46.1

Supplementary Figure 1 shows how the σ_x and σ_y of the 250 MeV symmetrically focused, asymmetrically focused and unfocused VHEE beams change with depth into the water phantom. This shows how the asymmetric beam has a much wider dose distribution at the entrance of the phantom, which produces a lower entrance dose at any point across the entrance of than phantom.

Supplementary Figure 2 shows how the σ_x and σ_y of the 100 MeV, 150 MeV, 200 MeV and 250 MeV asymmetrically focused VHEE beams change with depth into the water phantom. This shows that the penumbral spreading decreases with beam energy, which is due to reduced scattering of the higher energy beams.

Supplementary Table 3 shows the position of maximum dose in the TOPAS MC simulations and the focal length predicted for the Twiss parameters in the x- and y-planes for each of the beam energies and final quadrupole strengths used. This shows that here, the position of maximum dose TOPAS MC simulations follows the pattern in the x-plane focal lengths from the Twiss parameters, with differences due to the additional beam scattering in the TOPAS simulation from the air and water resulting in a lower penetration than the vacuum Twiss parameter case.

Supplementary Figure 3 further illustrates the difference between the vacuum Twiss parameter focal length predictions (shown as the Elegant results) and the full TOPAS simulation with air and the water phantom. Also shown are minor differences between the TOPAS vacuum simulation and the Elegant Twiss parameter results, which is most likely due to small differences

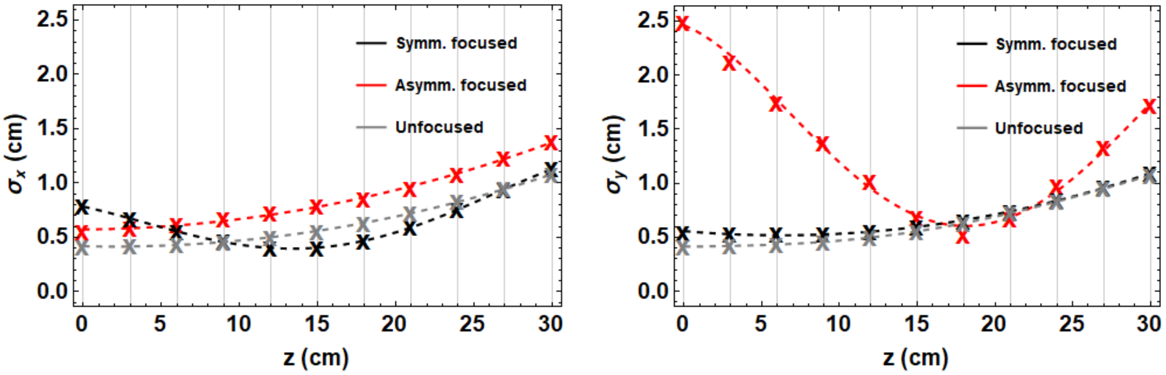

(a) σ_x vs depth through water phantom (b) σ_y vs depth through water phantom

Supplementary Figure 1. TOPAS-based Monte Carlo simulations of σ_x and σ_y vs depth through water phantom for asymmetrically, symmetrically and unfocused 250 MeV VHEE, all with initial σ= 0.4 cm.

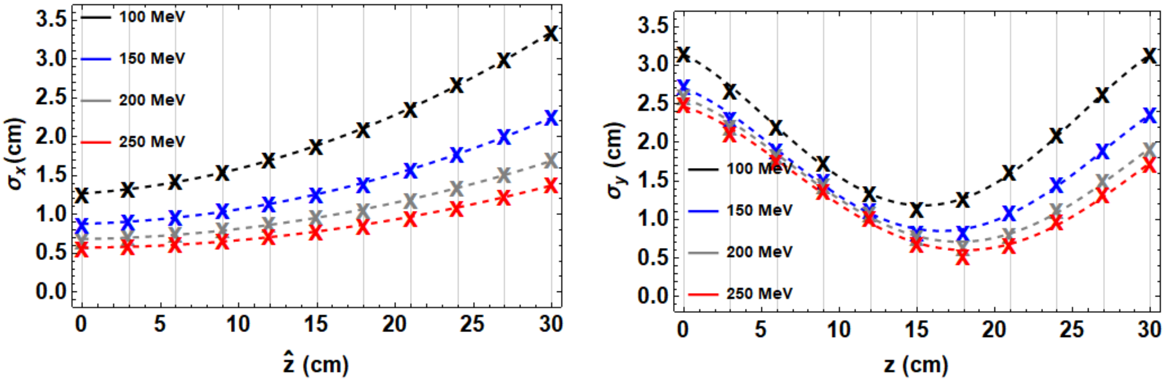

Supplementary Figure 2. σ_x and σ_y through the water phantom for each of the four beam energies and the magnet set-up shown in Supplementary Table S2 in Additional Materials.

in how the quadrupoles are modeled, as well as particle interactions in the TOPAS code that are not present in the Elegant simulations.

Supplementary Figures 4 and 5 show the transverse dose distributions of the SOEP and SOBP respectively. In both Figure 4 and 5, the colour is normalised to the maximum dose in the SOEP or SOBP. These Figures show the difference in dose distribution between the two types of spread-out peaks – the SOBP has a higher entrance dose, but the dose is contained within approximately 1 cm transversely, and there is no exit dose. The SOEP has a lower entrance dose, but spread out over a larger region, with the dose becoming more concentrated and contained over the target region, with a small exit dose. Both of these dose distributions could be used to treat a 3D tumour region, by combining multiple SOEPs or multiple SOBPs.

Supplementary Table 3. Positions of focal points L_{α_x}, L_{α_y} and position of maximum dose $\hat{z}$ for 100, 150, 200 and 250 MeV asymmetrically focused beams

Energy (MeV)	g_4 (T/m)	$\hat{z}$ (cm)	L_{α_x} (cm)	L_{α_y} (cm)
100	5.7	14.4	20.3	19.6
	6.0	12.3	16.9	25.2
	6.3	10.8	14.0	32.3
	6.6	8.7	11.4	41.6
	6.9	6.9	9.2	54.1
	7.2	5.4	7.1	72.1
	7.5	3.9	5.3	100.0
150	8.0	18	25.1	14.1
	8.3	17.1	22.4	16.9
	8.6	15.9	19.9	20.2
	8.9	14.1	17.6	23.9
	9.2	12.9	15.6	28.2
	9.5	11.4	13.7	33.2
	9.8	9.9	12.0	39.3
	10.1	8.7	10.4	46.7
	10.4	7.5	8.9	55.8
	10.7	6.3	7.6	67.5
	11.0	5.1	6.3	82.9
200	10.2	21.0	28.8	11.2
	10.8	19.5	24.2	15.0
	11.4	17.1	20.3	19.6
	12.0	14.4	16.9	25.2
	12.6	12.0	14.0	32.3
	13.2	9.9	11.4	41.6
	13.8	7.8	9.2	54.1
	14.4	6.0	7.1	72.1
	15.0	4.2	5.3	100.0
250	13.1	22.5	24.4	12.9
	13.7	19.8	23.1	16.2
	14.3	17.4	20.0	20.0
	14.9	15.3	17.3	24.4
	15.5	13.2	14.9	29.8
	16.1	11.4	12.8	36.3
	16.7	9.6	10.8	44.5
	17.3	7.8	9.0	55.1
	17.9	6.3	7.4	69.3

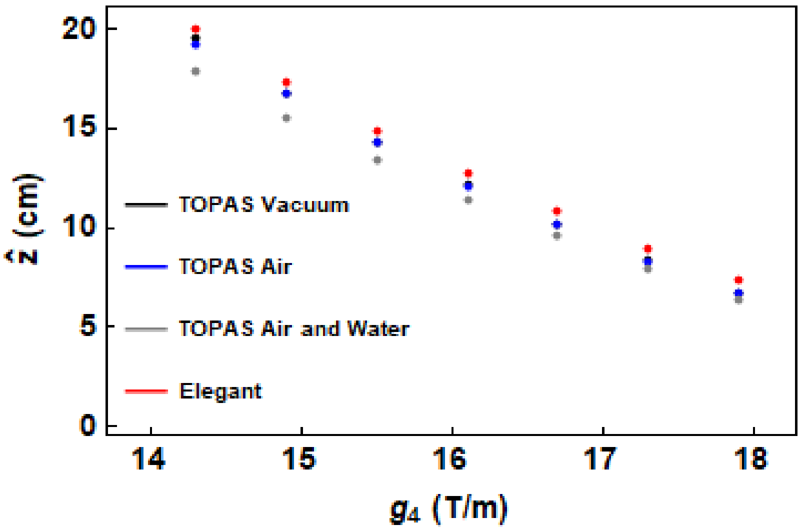

Supplementary Figure 3. Depth of maximum dose vs magnet strength for 250 MeV asymmetrically focused VHEE using the quadrupole strengths shown in Table 1 for TOPAS simulations in air, vacuum and the full water phantom simulation, with the analytical Twiss parameter predictions from Elegant shown. Note vacuum and air TOPAS results are the same to within 0.3 cm.

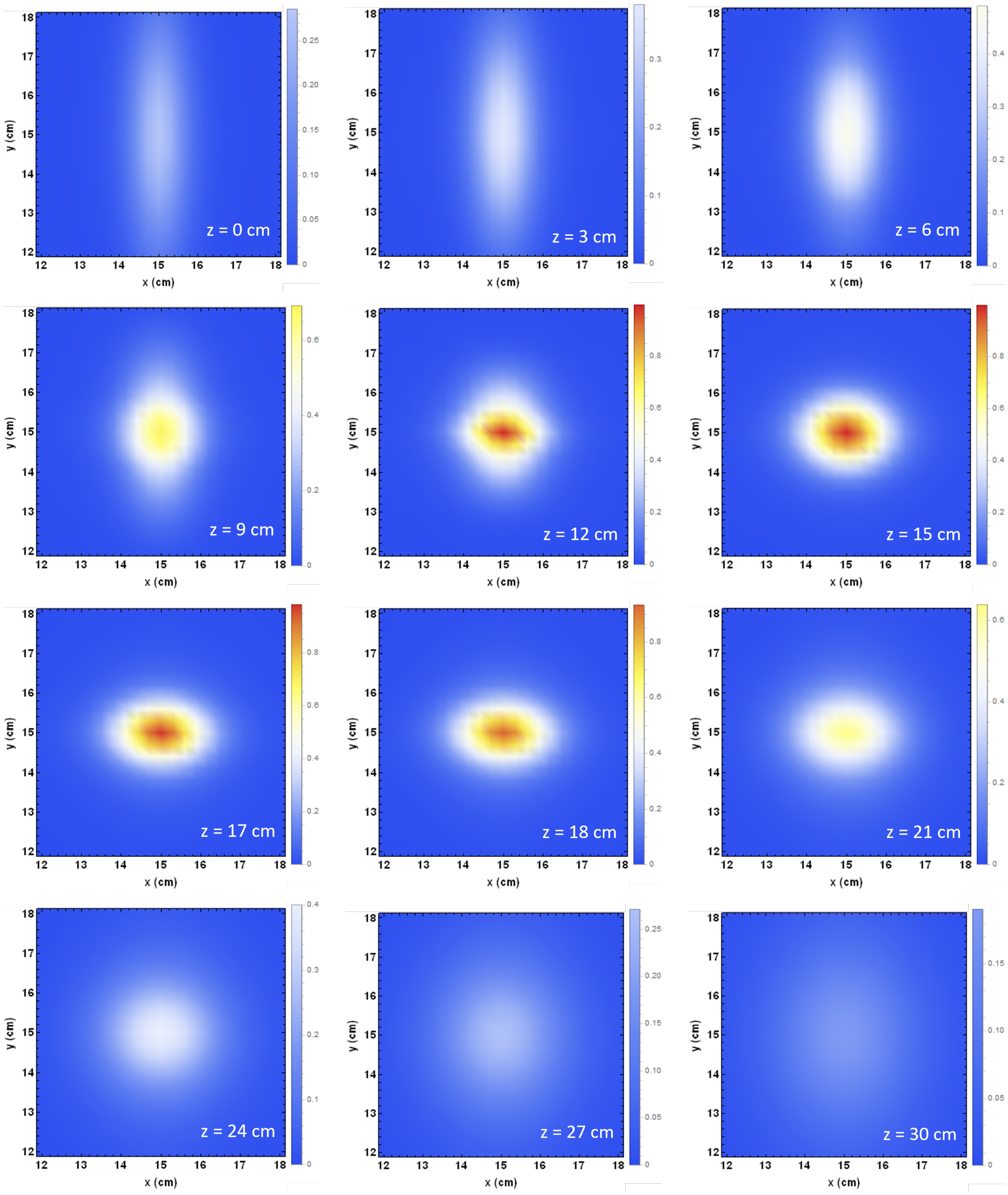

Supplementary Figure 4. Monte Carlo TOPAS simulations showing the transverse dose distribution of the SOEP in Figure 7 in the main article at depths into the phantom from 0-30 cm, in increments of 3 cm, with the edge of the target at 17 cm also shown.

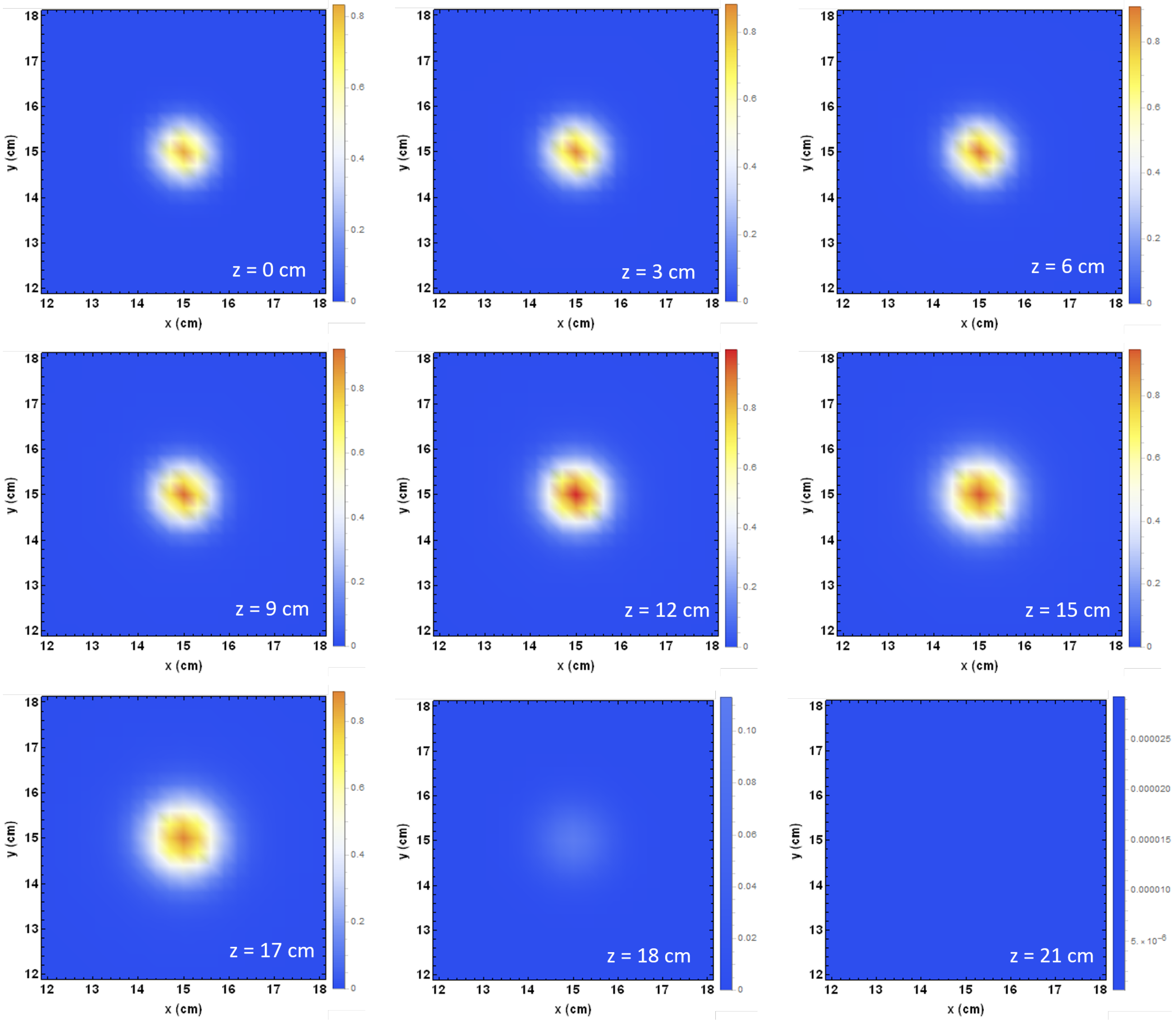

Supplementary Figure 5. Monte Carlo TOPAS simulations showing the transverse dose distribution of the SOBP in Figure 7 in the main article at depths into the phantom from 0-21 cm, in increments of 3 cm, after which the dose is negligible. The edge of the target at 17 cm is also shown.
